# DNA damage in different *Eisenia andrei* coelomocytes sub-populations after in vitro exposure to hydrogen peroxide

**DOI:** 10.1186/s40064-016-1950-x

**Published:** 2016-03-09

**Authors:** Laura Mincarelli, Costantino Vischetti, John Craft, Luca Tiano

**Affiliations:** Institute of Cancer Sciences, University of Glasgow, Glasgow, UK; Environmental, Food and Agricultural Sciences Department, Polytechnic University of Marche, Ancona, Italy; Biological and Biomedical Science Department, School of Health and Life Science, Glasgow Caledonian University, Glasgow, UK; Department of Clinical and Dental Sciences, Polytechnic University of Marche, Ancona, Italy

**Keywords:** Biomarker, Genotoxicity, Earthworm

## Abstract

Earthworms play an essential role in providing soil fertility and may represent an important soil contamination bio-indicator. They are able to ingest soil particles, adsorb substances throughout the intestinal epithelium into the coelomic cavity, where chemicals can come in direct contact with coelomic fluid. Earthworm coelomic fluid shelters leucocytes (coelomocytes) that differ significantly both structurally and functionally. Cellular variability could lead to different susceptibility towards contaminants possibly present in soil ecosystem. In order to define population specific dose response to chemicals and to identify a homogeneous cell population to be used as a relevant biomarker, we investigated different coelomocytes subpopulation, obtained by Percoll density gradient centrifugation (5–35 %), exposed ex vivo to H_2_O_2_ in the range of concentration 15–120 µM. DNA damage levels were assessed by the comet assay on unseparated coelomocytes and on three enriched cellular fractions (light, medium and heavy density subpopulations). All tested samples showed a dose–response genotoxic effect following H_2_O_2_ exposure. Moreover, light density sub-population appeared more susceptible to oxidative insult highlighted by a significant increase in DNA damage indexes at lower concentrations of H_2_O_2_. Present data suggested that in these experimental condition coelomocytes light fraction may represent a more sensitive biomarker of genotoxic insult.

## Background

Soil invertebrates are known to be efficient accumulators of xenobiotics, and to respond to their exposure in a sensitive and measurable manner, therefore they are widely employed as bioindicators of soil contamination (Cesar et al. 2015; Panzarino et al. 2015; Ji et al. 2013).

Among soil organisms, earthworms deserve particular interest because of their ecological role in soil biocenosis (Svendnsen et al. 2004). In fact, earthworms are common in a wide range of soil types and may represent 60–80 % of the total biomass (Bouchè et al. 1992). Finally, thanks to their ability to ingest soil particles they represent an extremely pertinent bio-indicator (Vernile et al. 2007).

*Eisenia andrei* Bouché (Lumbricidae) is one of the species commonly used in standard procedures defined by international protocols to assess acute toxicity of chemicals (OECD 1984, 2000) as well as sublethal effects (Reinecke and Reinecke 2004).

Standard toxicity tests evaluate parameters such as mortality, growth and reproduction, and are widely used in toxicant effects assessment (Reinecke et al. 2004). However, at lower level, cellular and molecular mechanisms might be disturbed, without an immediate impact on the organism physiology. Therefore molecular biomarker may provide complementary information regarding organism’s toxicants exposure response (Lourenço et al. 2011; Li et al. 2015; Velki et al. 2014). In particular, genotoxicity of different xenobiotics (e.g. pesticides, heavy metals) may trigger damages at cellular and eventually tissue and organism level (Li et al. 2009; Lourenço et al. 2011).

The comet assay has been widely accepted as a simple, sensitive and rapid tool for assessing DNA damage in individual eukaryotic cells (Dhawan et al. 2009) and it is widely used in ecotoxicology to detect DNA strand breaks (Tice et al. 2000).

In *Eisenia* species the comet assay is commonly conducted on extruded coelomocytes, a heterogeneous population of cells involved in various aspects of cellular and humoral immunity.

Coelomocytes from *Eisenia foetida* demonstrated increased DNA damage level following earthworm exposure both in vitro (Reinecke and Reinecke 2004; Di Marzio et al. 2005) and in polluted soil samples (Salagovic et al. 1996; Xiao et al. 2006; Rajaguru et al. 2003; Bustos-Obregon and Goicochea 2002; Zang et al. 2001).

Coelomocytes classification is largely based on differential staining, ultrastructure and granule composition. Kauschke et al. described four coelomocytes subpopulations: basophils, acidophils, neutrophils and chloragocytes (Kauschke et al. 2001). Basophils are identifiable by their dark-blue basophilic cytoplasm and their eccentrically located nuclei; acidophils instead contain numerous red–orange granules and neutrophils have a large centrally located nuclei and light colored cytoplasm when stained with Wrigth stain (Kauschke et al. 2001). Chloragocytes are modified peritoneal cells lying around the intestine and characterized by the presence of cytoplasmatic granulations, the chloragosomes (Joris 2000). Calisi et al. indentified a further subpopulation, granulocytes, that appears as large cells with pseudopodial process to Diff-Quick stain (Calisi et al. 2009).

Basophils, acidophils and neutrophils are involved in cell to cell recognition and phagocytosis, while chloragocytes are responsible for the synthesis and secretion of lytic factor (Cooper et al. 2002).

Several studies focus on earthworm immunity from the dual perspective of both immune system function and ecological importance. Immune system response has been indeed used as markers to evaluate effects of xenobiotic on the environment; moreover studies show that environmental xenobiotics can affect coelomocytes morphology and function (Calisi et al. 2009).

In the present study the responses of subpopulations of isolated coelomocytes to an in vitro stress (H_2_O_2_ as DNA damaging model), were analyzed, in order to investigate cellular fraction specific susceptibility and eventually identify a more sensitive subpopulation to be used as a high-sensitivity biomarker in environmental risk assessment.

## Methods

### Chemicals and reagents

Hydrogen peroxide (99 % purity), low melting agarose, normal melting agarose, NaCl, Na_2_EDTA, Tris–HCl, phosphate saline buffer, Triton X-100, Dimethyl sulfoxide (DMSO), were obtained from Sigma Aldrich. Solutions were freshly prepared in deionised water (18 MΩcm). TrevigenHT slides (Trevigen, Gaithersburg, MD, USA) were used for the comet assay.

### Earthworms

Earthworms (*Eisenia andrei*) were obtained from Lombricoltura Compagnoni farm, Lecco, Italy, and acclimated in the laboratory at 20 ± 2 °C for 36, 8 h light/dark, in organic compost. A homogeneous group (n = 75) of adults with a well-developed *clitellium* and an average weight of 379 ± 47 mg were collected from the breeding stock. Selected earthworms were washed with deionised water, and maintained for 24 h in glass vials (containing moistened Whatman No. 1 filter-paper) to clear their gut from soil residues before cell collection.

### Coelomocytes collection

Ethanol extrusion method was applied to collect worm coelomocytes. This method has been shown to provide highest cell viability and best separation among subpopulations, compared to electrical and ultrasound extrusion (Vernile et al. 2007).

Ethanol extrusion was carried out according to Eyambe et al. 1991, with slight modifications; Animals were individually incubated in a 5 % ethanol solution, for 3 min, at room temperature. Coelomocytes were spontaneously secreted in the extrusion solution and washed once in PBS 1× by centrifugation (400*g*, 10 min, room temperature) prior to cell separation. Coelomocytes extruded from 5 individuals were pooled together, washed, isolated as described below and finally used in the exposure assay. A total of 30 organism has been used, for a total of 6 different experiment replicates.

### Coelomocytes separation by density gradient centrifugation

**Fig. 1:**
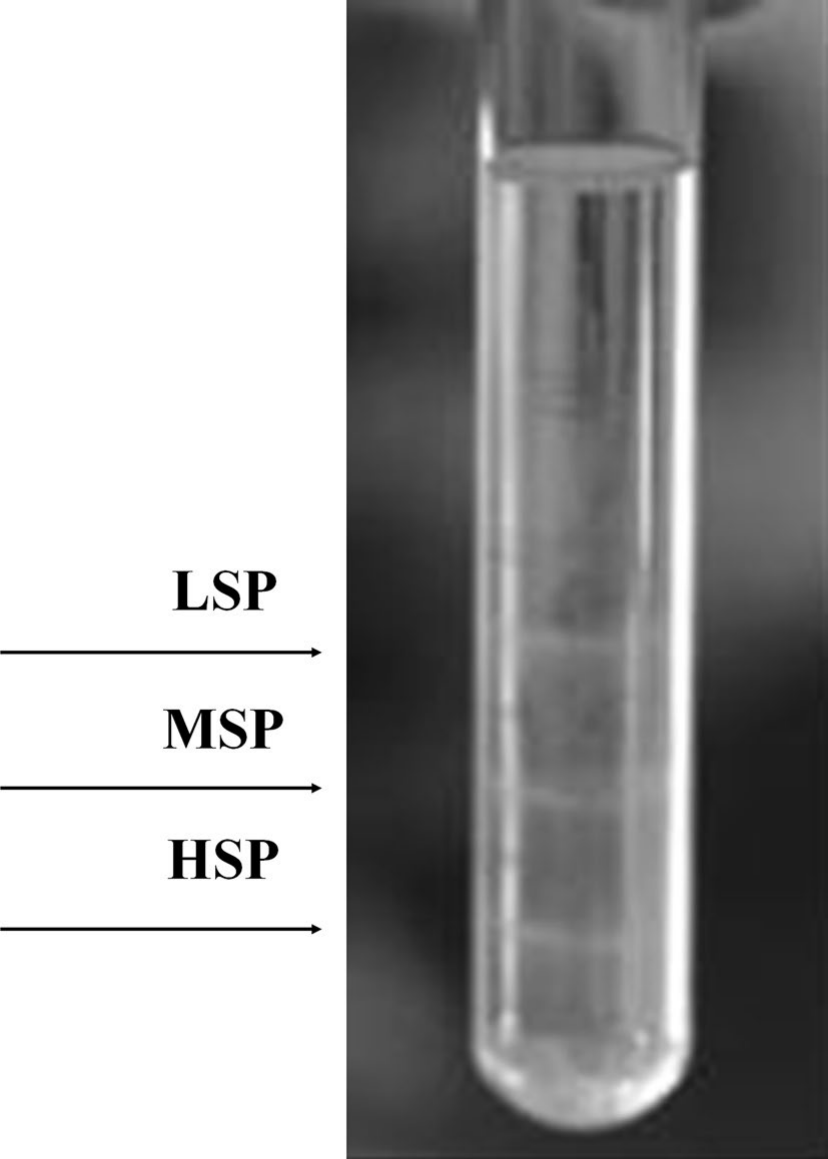
*E. andrei* coelomocytes separation by a Percoll gradient. Isolated fractions (LSP, MSP, HSP) shown. *LSP* 15 % Percoll cells bands, *MSP* 25 % Percoll cells band, *HSP* 35 % cells band

Coelomocytes separation was performed using Kauschke modified protocol (Kauschke et al. 2001). Coelomocytes suspensions were transferred onto a Percoll gradient made by carefully layering in a glass tube 2 ml of three Percoll solutions, at different densities, using a peristaltic pump. Concentrations employed were relative to 35 %–25 %–15 % Percoll in PBS (1×, 4 °C). Eventually, each Percoll gradient tube contained 6 ml of Percoll solution, stratified in three layers of 2 ml each. Cellular suspension (1.8–2 ml) at a concentration of 1.10^6^ cells/ml was finally layered on top and tubes were centrifuged at 400*g* for 20 min, 4 °C using a swing-out bucket, no brake was applied. Three distinct bands of cell sub-population were obtained at the boundaries between two gradients: heavy (35 %) medium (25 %) and light (15 %) (Fig. 1).

Separated bands were collected using a Pasteur pipette and washed in PBS 1× (400 g, 10 min, 4 °C).

Viability was assessed by means of Trypan Blue assay and was over 80 % for all collected fractions.

### In vitro exposure

Total un-separated population and the three density separated sub-populations were exposed to increasing concentrations of H_2_O_2_ in the range 15–120 µM diluted in PBS at a concentration of 2 × 10^5^ cells/ml in 250 µl H_2_O_2_. Cells were incubated in the dark, at 4 °C for 1 h, washed in PBS by centrifugation at 400 g, 10 min, 4 °C and immediately used to assess DNA damage by comet assay.

Five independent separation and exposure experiments were performed.

### Comet assay

DNA strand breaks were assessed using the alkaline version of the comet assay (pH 13), which allows detection of single and double strand breaks and alkali labile sites. DNA damage indexes (Tail length, Tail intensity and Tail moment) were assessed by computer aided image processing, using a custom made software developed in our laboratory (Tiano et al. 2012).

Five independent experiments were performed using freshly extruded cells each time. Each condition was analyzed by three independent technical replicates.

### Slide preparation

Washed cells collected by centrifugation, resuspended in 0.7 % Low melting agarose (LMA) at 37 °C, and stratified on HT trevigen slides. Each slide was able to host 20 spots of cell containing agarose, separated by silicone barriers. Each spot was produced by layering 15 µl of low melting agarose containing 5000 cells each sample was stratified in triplicate. After LMA solidification on ice (4 °C, dark, 10 min) slides were submerged in an alkaline lysis solution (pH 10) containing NaCl (2.5 mol/L), disodium salt EDTA (0.1 mol/L), Tris–HCl (10 mmol/L), DMSO 10 %, Triton X-100 1 %, and kept at 4 °C for at least 1 h.

### Electrophoresis

Prior to electrophoresis, the slides were incubated for 20 min in the dark at 4 °C in alkaline electrophoresis buffer (1 mmol/L EDTA and 300 mmol/L sodium hydroxide, pH 13) to unwind DNA and to express alkali labile sites as single-strand breaks. Finally nucleoids were electrophoretically processed under alkaline (pH 13) conditions to produce comets at 11 V/cm, neutralized in Tris–HCl pH 7.5 for 5 min, dehydrated in methanol 70 % for 2 min, dried at 60 °C for 2 min and stored for microscopic examination. Before analysis, slides were stained with ethidium bromide (20 µg/mL) and viewed under fluorescent light using an Olympus BX51 fluorescence microscope (Olympus, Tokyo, Japan) connected to a PC. Observations were performed at a magnification of 200×.

### Computer-aided comet analysis

30 to 50 randomly acquired images of microscopic fields relative to each sample were recorded to enable analysis of 150 cells for each triplicate. Images were processed using custom-made software that enables automatic identification of the comets, greatly reducing operator-dependent variability. The DNA damage extent has been quantified using Tail intensity, TI, (corresponding to Tail DNA %) Tail length, TL (extent of migration expressed in µm) and Tail moment, TM, whose value is affected by both TL and TI, as parameters.

### Statistical methods

Results for the considered comet indexes (Tail length, Tail Intensity and Tail moment) are expressed as means of the median and 75th percentile ± standard deviation (SD).

Normal distribution of the data was verified using Kolmogov–Smirnov test and consequently significant differences among groups has been estimated using ANOVA one way test (Duez et al. 2003) and LSD post hoc test. Minitab software has been used to perform statistical analysis.

Analysis has been performed within each enriched subpopulations group and comparing each group (UP, LSP, MSP, HSP) toward UP0, to detect any significant DNA damage level with respect to the un-separated unexposed populations of cells.

## Results

### Comet assay

**Fig. 2:**
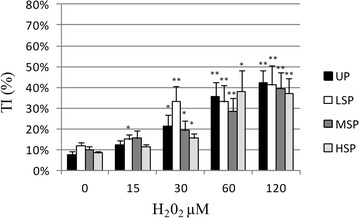
DNA damage (median TI). Induction of DNA damage (median TI) in *E. andrei* coelomocytes following in vitro exposure to increasing concentrations H_2_O_2_. The values are mean ± SEM; *significant difference from unexposed total population (*p < 0.05; **p < 0.01). *UP* unseprated total population, *LSP* light cell subpopulation, *MSP* medium cells subpopulation, *HSP* heavy cell subpopulation

**Fig. 3:**
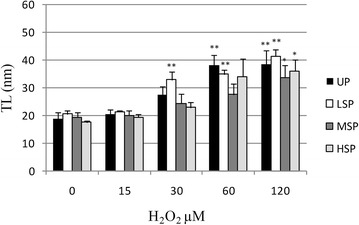
DNA damage (median TL). Induction of DNA damage (represented as median TL) in *E. andrei* coelomocytes following in vitro exposure to increasing concentrations H_2_O_2_. The values are mean ± SEM; *significant difference from unexposed total population (*p < 0.05; **p < 0.01). *UP* unseprated total population, *LSP* light cell subpopulation, *MSP* medium cells subpopulation, *HSP* heavy cell subpopulation

**Fig. 4:**
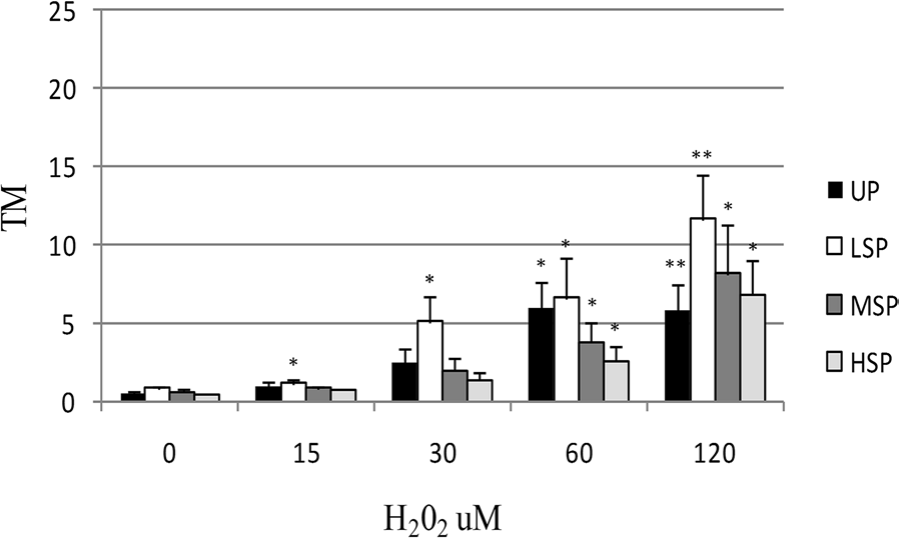
DNA damage (median TM). Induction of DNA damage (represented as median TM) in *E. andrei* coelomocytes following in vitro exposure to increasing concentrations H_2_O_2_. The values are mean ± SEM; *significant difference from unexposed total population (*p < 0.05; **p < 0.01). *UP* unseprated total population, *LSP* light cell subpopulation, *MSP* medium cells subpopulation, *HSP* heavy cell subpopulation

**Fig. 5:**
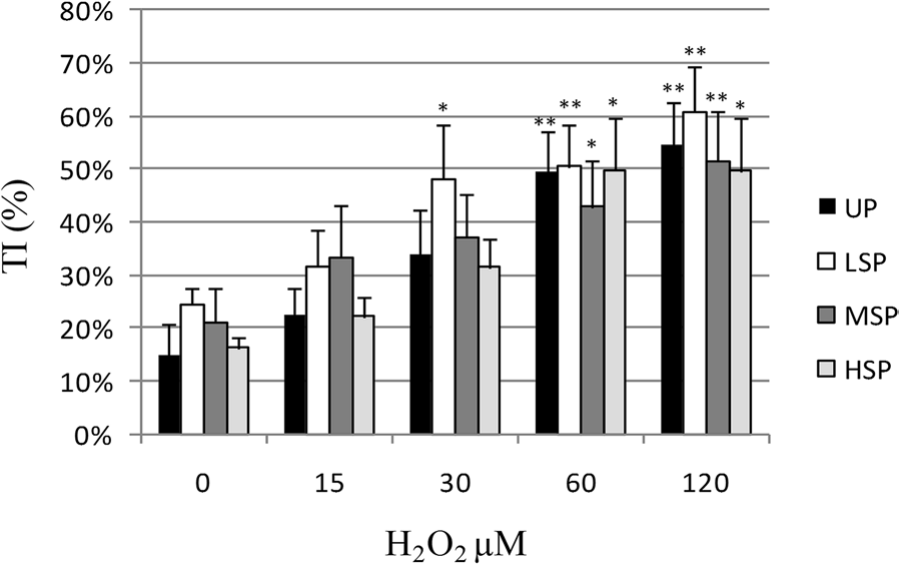
DNA damage (75th percentile TI). Induction of DNA damage (represented as 75th percentile TI) in *E. andrei* coelomocytes following in vitro exposure to increasing concentrations H_2_O_2_. The values are mean ± SEM; *significant difference from unexposed total population (*p < 0.05; **p < 0.01). *UP* unseprated total population, *LSP* light cell subpopulation, *MSP* medium cells subpopulation, *HSP* heavy cell subpopulation

As reported in Figs. 2, 3, 4 and 5 extruded coelomocytes directly exposed to increasing concentration of H_2_O_2_ produced a significant elevation of all tested DNA damage indexes (Tail intensity, TI, Tail moment, TM, and Tail length, TL). In particular, H_2_O_2_-induced DNA damage was shown to be dose dependent in all considered cell populations.

For what concerns unexposed control cells, un-separated total population (UP0) presented comparable levels of DNA damage as the Percoll-separated cells, suggesting that density gradient centrifugation did not alter DNA fragmentation and also that baseline fractions were homogenous in relation to their DNA oxidative status. Therefore, UP0 cells were used as baseline value.

Figure 2 shows the distribution of the data according to the Tail intensity (TI), that is proportional to the percentage of damaged DNA. The extent of DNA damage reached a significant level at 30 µM H_2_O_2_ in the total population (UP; +14 %, p < 0.05).

When considering subpopulations, MSP behaved similarly to the un-separated population (+12 %, p < 0.05); on the contrary LSP showed a higher sensitivity to oxidative insult producing a significant increase of Tail intensity already at 15 µM H_2_O_2_ (+7 % p < 0.05). On the other hand HSP fraction resulted the less sensitive to oxidative insult: 30 μM H_2_O_2_ was significantly inducing DNA damage in this cell type, but to a lower extent (+9 %, p < 0.05). Moreover, while in all the other populations the level of damage produced by H_2_O_2_ reaches highly significant levels already at 60 µM, HSP cells show highly significant increases in DNA damage only in cells exposed to 120 µM H_2_O_2_ (+30 %, p < 0.01).

Another index of DNA damage is represented by the length of the comet TL, reported in Fig. 3. This index is more prone to saturation and hence its correlation with the DNA damage is more stringent at lower levels of damage.

Distribution of data according to TL also confirmed what observed for the TI: This parameter as well showed an increased susceptibility of LSP already at 30 µM H_2_O_2_ (+75 %, p < 0.01), while DNA damage in UP was significantly modified only starting at 60 µM H_2_O_2_ (+101 %, p < 0.01). In that case however both MSP and HSP showed a significant increase of median values of TL only when exposed to the highest H_2_O_2_ concentration.

Finally considering Tail moment (TM) median values, an index that is influenced both by Tail intensity and Tail length, a higher susceptibility of LSP was confirmed producing a significant level of increase of this index already at 15 µM H_2_O_2_ (+143 %, p˂0.05) reaching an highly significant difference in presence of 120 µM H_2_O_2_ (Fig. 4). On the contrary UP as well as MSP and HSP subpopulations did not show a significant increase in median TM for exposures at concentrations lower than 60 µM H_2_O_2_.

In an attempt to better describe susceptibility to the genotoxic insult we also evaluated the distribution of highly damaged cells (Fig. 5), indicated by the 75th percentile values of Tail intensity over the analyzed population that are summarized in Fig. 5. A Significant increase of 75th percentile TI values over base line values was observed in LSP cells exposed to 30 µM H_2_O_2_ (+33 % LSP, p < 0.05). In presence of 60 µM H_2_O_2_ UP and LSP damage was highly significant over control (UP0) values (+35 % UP, p < 0.01; +35 % LSP, p < 0.01). Finally, also in this case HSP confirmed a higher resistance to DNA damaging effect of hydrogen peroxide as only H_2_O_2_ 120 µM produced a significant increase in highly damaged cells (+35 %; p < 0.05), while the elevation for all the other fractions presented a higher level of significance (+46 % LSP; +40 % UP; +37 % MSP, p < 0.01).

## Discussion

DNA is an important target of environmental stress in both aquatic and terrestrial organism (Reinecke and Reinecke 2004) and DNA strand breakage has been proposed as a sensitive indicator of genotoxicity (Lourenço et al. 2011).

Comet assay is a simple and reliable technique to assess DNA damage on the basis of altered electrophoretic mobility of DNA, at single cell level, that has received important attention over the last decade and has been shown to be a useful tool also in environmental risk assessment (Langie et al. 2015; Costa et al. 2014). Earthworms represent an interesting animal model and a useful candidate organism in soil ecotoxicology studies due to its direct exposure to soil contaminants.

In earthworms, indeed, exchange of gas, fluids and nutrients between the organism itself and its environment is almost direct.

Not presenting distinct respiratory organs, gas exchange occurs through the thin epithelium separating endodermal tissue from the soil. Moreover, internally the intestine is separated from the endoderm by the coelomatic cavity. Coelome has several physiological functions in *Anellida* including those of hydrostatic support and coadiuvate locomotion. It is therefore of extreme relevance to organism health that earthworms ingest soil that comes in direct contact with coelomic fluid and herein present immune cells, so called coelomocytes.

Since coelomic fluids contains coelomocytes which differ in their structure and function (Bilej et al. 1990; Cooper 1986), it is possible that their variability could lead to different susceptibility to pollutants.

Xenobiotic alterations on coelomocytes morphology and functionality have been investigated at subpopulation level by means of bright field microscopy investigation and flow cytometric studies. In particular modification in distribution of coelomocytes subpopulation has been evaluated following animals long term incubation in polluted soil (Vernile et al. 2007) and pollutants-induced morphometric alteration in granulocyte has been also assessed (Calisi et al. 2009).

However, despite the acknowledged relevance of the comet assay in coelomocytes, no studies have been reported on isolated subpopulations.

In the present study the density gradient was performed to separate three different coelomocytes enriched subpopulations. Subpopulation LSP (15 % Percoll band), according to Kauschke et al. contained mostly neutrophilc cells (Kauschke et al. 2001); the second band, (25 % Percoll) was rich in basophils and also contains some small chloragocytes, while the lowest band, at (35 % Percoll band) contained mostly chloragocytes (Kauschke et al. 2001). Acidophil were parceled out in all the bands (Kauschke et al. 2001).

Coelomocytes were sensitive to the DNA-damaging agents, showing a dose–response effect.

Results should be interpreted taking into account that Tail length is more sensitive to lower levels of DNA damage but its values are rapidly saturated as DNA damage increases (Collins et al. 2008). Tail intensity, on the contrary is considered a better indicator of DNA damage over a wide range of concentrations of insult since its value might virtually range from 0 to 100 %. Tail moment has been therefore indicated as a useful synthetic index since its levels are influenced both by TL and TI. Comparative analysis of samples shows, beside an overall oxidant dose dependent induction of DNA damage, a marked enhanced response of the LSP and a duller response of the HSP.

The present report represents, to the best of our knowledge, the first report of the comet assay applied to a fractionated population of earthworm coelomocyte. Coelomocytes separation using a discontinuous Percoll gradient allowed to highlight a light density population of cells that are apparently more susceptible to DNA damage. Increase susceptibility might depend on lowered DNA repair mechanisms or enhanced sensitivity to extracellular stimuli. Moreover the heavy density population of coelomocytes resulted, on the contrary, more resistant probably due to analogue reasons.

The described features could be efficiently exploited in order to extend the range of sensitivity of *E. andrei* when used as ecotoxicological bioindicator.

In fact, summarizing, we propose that analysis of a fractioned coelomocytes population response to genotoxic stimuli might provide a more robust information of their biological response to stress, enabling detection of effects at very low doses of stressors using the low density enriched population LSP while analysis of isolated enriched high density fraction HSP might enable comparison of biological responses in highly contaminated environments.

The present study was focused on the different threshold of sensitivity of the cells and therefore a standard chemical used as a positive control in genotoxicity study, such as hydrogen peroxide, was used. Nevertheless future development of this experimental model could enable its application to xenobiotic contaminated soils both in the laboratory and in the field studies.

## Conclusions

In conclusion, density gradient separation of enriched subpopulation in low density and high density coelomocytes provides a useful methodology to identify cell fraction showing different susceptibility to genotoxic insult. Cell fraction analysis provides more accurate information as opposed to the unseparated coelomic population. Isolated subpopulation analysis could therefore represent an interesting biomarker of chemicals genotoxicity, allowing an early analysis and a more accurate identification of genotoxicity hazard level of a contaminant.

Further investigations are required to validate these data using an in vivo exposure model. Moreover, further separation steps in Percoll media could be necessary to isolate coelomocytes subpopulations to a higher level of homogeneity.
